# Case Report: Late paradoxical intrapulmonary shunt after endoscopic lung volume reduction with endobronchial valves for severe emphysema

**DOI:** 10.3389/fmed.2025.1572900

**Published:** 2025-05-15

**Authors:** Jacques Dzuko Kamga, Christophe Gut Gobert, Thomas Egenod, Nicolas Guibert, Pierre-Yves Le Roux

**Affiliations:** ^1^Department of Nuclear Medicine, Brest University Hospital, Brest, France; ^2^Department of Pneumology, Brest University Hospital, Brest, France; ^3^GETBO, INSERM, UMR1304, Université de Bretagne Occidentale, Brest, France; ^4^Department of Pneumology, Limoges University Hospital, Limoges, France; ^5^Department of Pneumology, Toulouse University Hospital, Toulouse, France

**Keywords:** endo-bronchial valves, endoscopic lung volume reduction (ELVR), emphysema, ventilation-perfusion SPECT–CT, lung scintigraphy, pulmonary shunt

## Abstract

Endoscopic endobronchial valve lung volume reduction in the management of severe emphysema reduces volume inflation in the most severely affected areas, with the hope of clinical improvement. We report the case of a patient who presented with late progressive respiratory deterioration following endoscopic endobronchial valve lung volume reduction. Ventilation/perfusion (V/Q)-SPECT/CT showed unexpectedly high perfusion in the atelectasis of the treated lobes, reflecting a paradoxical intrapulmonary shunt. This paradoxical complication of endobronchial valve treatment for severe emphysema is rare and highlights the potential usefulness of performing V/Q-SPECT/CT imaging in patients presenting with respiratory deterioration following endoscopic lung volume reduction.

## Introduction

Endoscopic lung volume reduction (ELVR) in the treatment of severe emphysema reduces pulmonary inflation in the most affected areas (target lobes), aiming to achieve clinical improvement (1). This method provides a less invasive alternative to lung volume reduction surgery, developed by Cooper et al. (2, 3). Studies have shown that lung volume reduction via endobronchial valves in carefully selected patients, with low collateral ventilation, leads to functional benefits, such as improvements in forced expiratory volume in 1 s (FEV1), 6-min walk test (6MWT) performance, and a reduction in residual volume (RV), in addition to improvements in quality of life (4–7). Although early complications have been documented, little information is available regarding late paradoxical phenomena (8–10). This report presents a case where a paradoxical intrapulmonary shunt was detected several months after treatment.

## Case description

**Figure 1:**
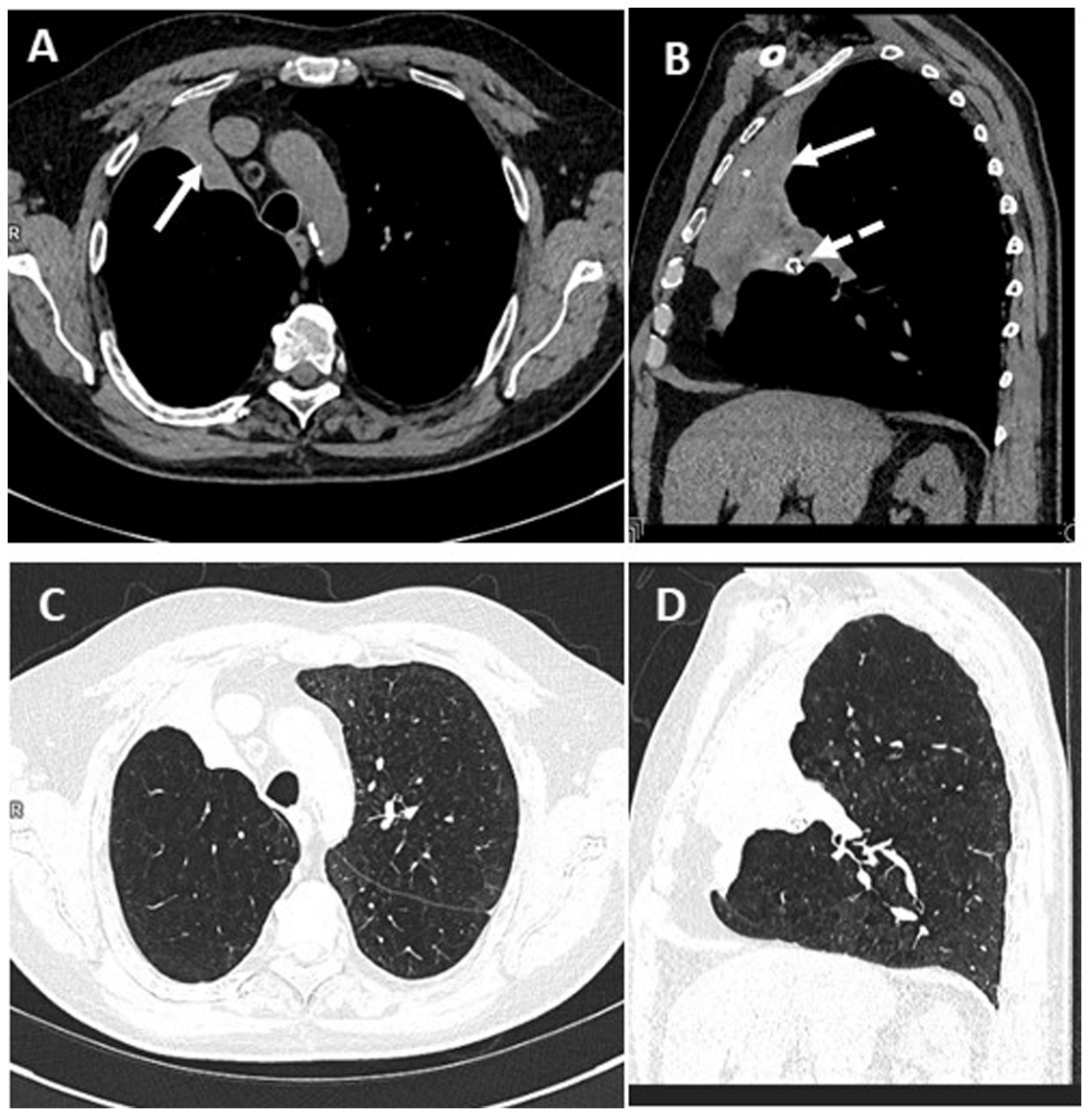
Axial and sagittal CT slices passing through the atelectasis, in mediastinal **(A,B)** and pulmonary **(C,D)** windows. The white arrow shows the atelectasis; the white dashed arrow shows a Zephyr endobronchial valve.

A 63-year-old man with severe emphysema underwent endoscopic lung volume reduction (ELVR) following functional assessment (Table 1—FEV1 at 34% of the predicted value, RV at 180% of the predicted value, 6MWT: 265 m) and arterial blood gas analysis (pH 7.44, pO_2_ 86 mmHg, bicarbonate 30 mmol/L, pCO_2_ 44 mmHg on 2 L of oxygen). Emphysema was diffuse, primarily localized in the apices of the lungs. The percentage of voxels with a density < −950 HU on a dedicated CT scan in the treated lobes (middle and right upper lobes) was 37% for the middle lobe (ML) and 45% for the right superior lobe (RSL). For the other lobes, the percentages were as follows: 32% for the right inferior lobe (RIL), 39% for the left superior lobe (LSL), and 35% for the left inferior lobe (LIL). The patient underwent a two-stage lung volume reduction using Zephyr^®^ endobronchial valves (Figure 1B—dashed white arrow). In the first stage, valves were placed in the B1, B2, B3, and B5 segments of the right upper and middle lobes. A second intervention, 6 weeks later, involved placing a valve in the B4 segment.

**Table 1:** Functional assessments of the patient and scintigraphic quantification of ventilation and perfusion before and after treatment.

	FEV1	RV	6MWT	%V target	%Q target
Baseline	0.94 L (34%)	180%	264 m	34	22
10 months after valve insertion	1.28 L (45%)	114%	295 m	0	2
18 months after valve insertion	1.25 L (44%)	114%	< 100 m	0	5

%V target, relative ventilation of the target lobes compared to the total lung ventilation; %Q target, relative perfusion of the target lobes compared to the total lung perfusion.

**Figure 2:**
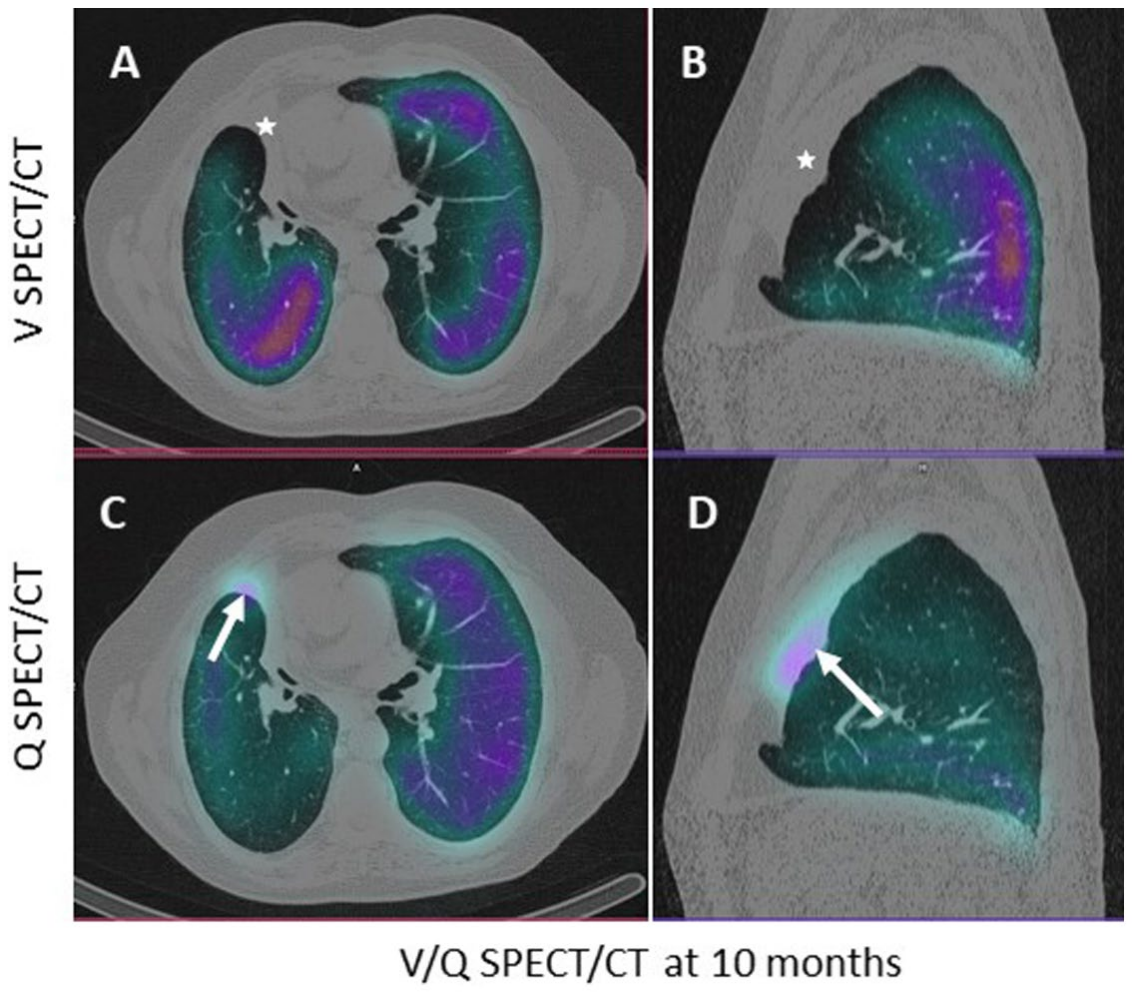
Axial **(A–C)** and sagittal **(B–D)** CT slices passing through the atelectasis, in pulmonary window. The white arrow shows the low (non-significant) uptake of macroaggregated albumin in the perfused atelectasis **(C,D)**; the white star shows the absence of ventilation **(A,B)**.

At 10 months, a CT scan revealed complete atelectasis of the treated lobes (Figures 1A,B, white arrows). A ventilation/perfusion scintigraphy (V/Q-SPECT/CT) conducted at that time showed no significant abnormalities in the atelectatic areas (low perfusion uptake of macroaggregated albumin (MAA) retrospectively—Figures 2C,D without associated ventilation—Figures 2A,B). The patient’s evaluation (Table 1) showed clinical improvement with a reduction in dyspnea and an improvement in FEV1 (45%) and RV (114%).

**Figure 3:**
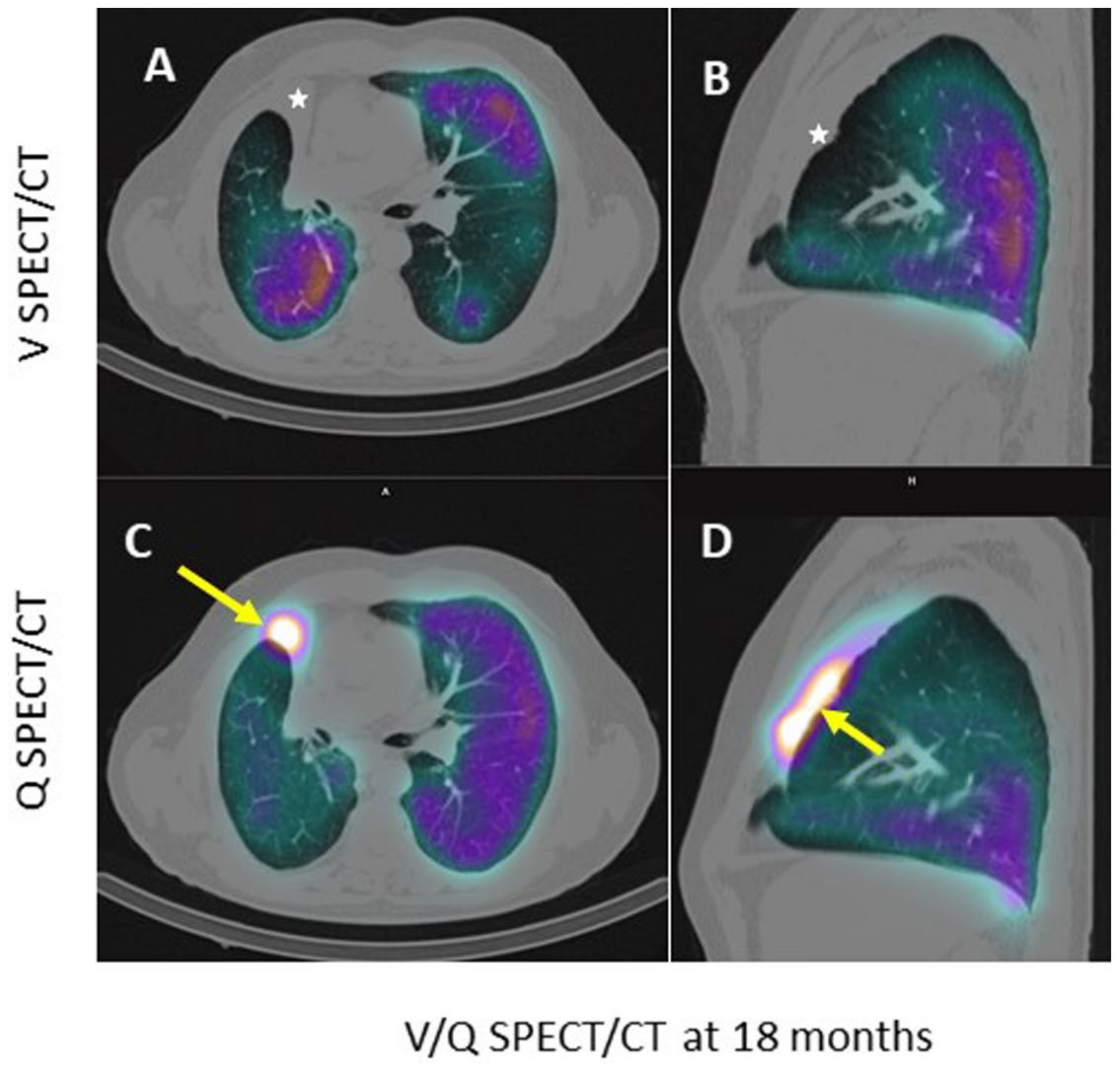
Axial **(A–C)** and sagittal **(B–D)** CT slices passing through the atelectasis, in pulmonary window. The white arrow shows the very high uptake of macroaggregated albumin in the perfused atelectasis **(C,D)**; the white star shows the absence of ventilation **(A,B)**.

However, at 18 months, a new V/Q-SPECT/CT, performed due to worsening respiratory status (dyspnea, cyanosis, and a reduction in the 6MWT to less than 100 m—Table 1), revealed abnormal and very high uptake of macroaggregates of albumin (MAA) (Figures 3C,D in the atelectatic, non-ventilated lobes—Figures 3A,B). Arterial blood gas at 24 months showed: pH 7.46, pO_2_ 66 mmHg, pCO_2_ 33 mmHg on 3 L of oxygen.

Relative lung ventilation and perfusion in the target lobes at M0, M10, and M18 are presented in Table 1. In the pre-therapeutic SPECT/CT evaluation, the target lobes (right middle and upper lobes) represented 30% of the total lung volume, 34% of the total ventilation, and 22% of the total perfusion, with 43% of the right lung perfusion. At M10 and M18, the relative lung perfusion in the target lobe increased from 2 to 5% (7 and 14% of the right lung perfusion) while the relative lung ventilation was 0%.

## Discussion

The unidirectional endobronchial valve directs airflow from the alveoli to the outside of the lung, causing volume reduction and atelectasis of the target lobes (1), thereby limiting pulmonary hyperinflation (1). Atelectasis of the target lobes causes hypoxemic vasoconstriction (suppression of perfusion) and vascular redistribution to the rest of the lung (11, 12). The patient was not on medications that would inhibit vasoconstriction, which could maintain perfusion to the atelectatic area (13). The significant uptake of MAA in this atelectasis, which is unusual in pulmonary scintigraphy, was particularly notable, indicating preferential pulmonary blood flow to this non-ventilated territory, consistent with an intrapulmonary shunt. The arterial blood gas at 24 months also showed a shunt effect, with a decrease in PaCO_2_ and PaO_2_ (partially corrected by increasing the O_2_ from 2 to 3 L/min to maintain a PaO_2_ of 66 mmHg, compared to 86 mmHg initially), resulting in PaO_2_ + PaCO_2_ < 120 mmHg. This late paradoxical phenomenon had not previously been described as a potential complication of the technique (13, 14). Ventilation/perfusion scintigraphy (V/Q-SPECT/CT), while useful for functional lung quantification and selection of target lobes prior to lung volume reduction (15, 16), is not widely performed during follow-up, which could explain the lack of awareness of this phenomenon. Due to the worsening of the respiratory status and no clear etiology despite cardiac evaluations (echocardiogram, electrocardiogram, and myocardial perfusion scintigraphy), the endobronchial valves were eventually removed at 24 months, but there was no improvement in respiratory function. Unfortunately, we did not perform late imaging after the valve removal, which would have provided a better understanding of the evolution of the V/Q mismatch and the re-expansion of the target lobes.

## Conclusion

This case illustrates an intrapulmonary shunt, a rare and paradoxical complication of endobronchial valve treatment for severe emphysema, and highlights the potential usefulness of performing V/Q-SPECT/CT in patients presenting with respiratory deterioration following endoscopic lung volume reduction.

## Data Availability

The original contributions presented in the study are included in the article/supplementary material, further inquiries can be directed to the corresponding author.

## References

[1] Mal H Bunel V Marceau A Dombret MC Debray MP Crestani B. Endoscopic lung volume reduction for emphysema. Rev Mal Respir. (2019) 36:880–8. doi: 10.1016/j.rmr.2019.05.039, PMID: 31208885

[2] Ingenito EP Wood DE Utz JP. Bronchoscopic lung volume reduction in severe emphysema. Proc Am Thorac Soc. (2008) 5:454–60. doi: 10.1513/pats.200707-085ET, PMID: 18453355 PMC2645319

[3] Cooper JD Trulock EP Triantafillou AN Patterson GA Pohl MS Deloney PA. Bilateral pneumectomy (volume reduction) for chronic obstructive pulmonary disease. J Thorac Cardiovasc Surg. (1995) 109:106–19. doi: 10.1016/S0022-5223(95)70426-4, PMID: 7815786

[4] Klooster K ten Hacken NH Hartman JE Kerstjens HA van Rikxoort EM Slebos DJ. Endobronchial valves for emphysema without Interlobar collateral ventilation. N Engl J Med. (2015) 373:2325–35. doi: 10.1056/NEJMoa1507807, PMID: 26650153

[5] Valipour A Slebos DJ Herth F Darwiche K Wagner M Ficker JH. Endobronchial valve therapy in patients with homogeneous emphysema. Results from the IMPACT study. Am J Respir Crit Care Med. (2016) 194:1073–82. doi: 10.1164/rccm.201607-1383OC, PMID: 27580428

[6] Kemp SV Slebos DJ Kirk A Kornaszewska M Carron K Ek L. A multicenter randomized controlled trial of Zephyr endobronchial valve treatment in heterogeneous emphysema (TRANSFORM). Am J Respir Crit Care Med. (2017) 196:1535–43. doi: 10.1164/rccm.201707-1327OC, PMID: 28885054

[7] Criner GJ Sue R Wright S Dransfield M Rivas-Perez H Wiese T. A multicenter randomized controlled trial of Zephyr endobronchial valve treatment in heterogeneous emphysema (LIBERATE). Am J Respir Crit Care Med. (2018) 198:1151–64. doi: 10.1164/rccm.201803-0590OC, PMID: 29787288

[8] Chakravorty S Mahajan AK. Complications following bronchoscopic lung volume reduction (BLVR). AME Med J. (2023) 8:31. doi: 10.21037/amj-23-106

[9] Franzen D Straub G Freitag L. Complications after bronchoscopic lung volume reduction. J Thorac Dis. (2018) 10:S2811–5. doi: 10.21037/jtd.2018.06.66, PMID: 30210835 PMC6129799

[10] Fiorelli A D'Andrilli A Bezzi M Ibrahim M Anile M Diso D. Complications related to endoscopic lung volume reduction for emphysema with endobronchial valves: results of a multicenter study. J Thorac Dis. (2018) 10:S3315–25. doi: 10.21037/jtd.2018.06.69, PMID: 30450237 PMC6204336

[11] Marshall BE. Importance of hypoxic pulmonary vasoconstriction with atelectasis. Adv Shock Res. (1982) 8:1–12. PMID: 7136935

[12] Ward JP McMurtry IF. Mechanisms of hypoxic pulmonary vasoconstriction and their roles in pulmonary hypertension: new findings for an old problem. Curr Opin Pharmacol. (2009) 9:287–96. doi: 10.1016/j.coph.2009.02.006, PMID: 19297247 PMC2692823

[13] Gompelmann D Eberhardt R Herth F. Endoscopic volume reduction in COPD - a critical review. Dtsch Arztebl Int. (2014) 111:827–33. doi: 10.3238/arztebl.2014.0827, PMID: 25556601 PMC4284519

[14] Gompelmann D Shah PL Valipour A Herth FJF. Bronchoscopic thermal vapor ablation: best practice recommendations from an expert panel on endoscopic lung volume reduction. Respiration. (2018) 95:392–400. doi: 10.1159/000489815, PMID: 29895029

[15] Tee VST Nguyen P Jersmann H Grosser D Crouch B Lorraine B. Use of ventilation-perfusion single-photon emission computed tomography to select the target lobe for endobronchial valve lung volume reduction. Respiration. (2021) 100:886–97. doi: 10.1159/000515336, PMID: 33774642 PMC8491486

[16] Le Pennec R Schaefer W Tulchinsky M Lamoureux F Roach P Rischpler C. Performance and interpretation of lung scintigraphy: an evaluation of current practices in Australia, Canada, France, Germany, and United States. Clin Nucl Med. (2024) 49:997–1003. doi: 10.1097/RLU.0000000000005396, PMID: 39086050

